# CD74 in Kidney Disease

**DOI:** 10.3389/fimmu.2015.00483

**Published:** 2015-09-23

**Authors:** Lara Valiño-Rivas, Ciro Baeza-Bermejillo, Laura Gonzalez-Lafuente, Ana Belen Sanz, Alberto Ortiz, Maria Dolores Sanchez-Niño

**Affiliations:** ^1^Instituto de Investigación Sanitaria de la Fundación Jiménez Díaz, Universidad Autónoma de Madrid, Madrid, Spain; ^2^Red de Investigación Renal (REDINREN), Madrid, Spain; ^3^School of Medicine, Universidad Autónoma de Madrid, Madrid, Spain; ^4^Fundacion Renal Iñigo Alvarez de Toledo-IRSIN, Madrid, Spain

**Keywords:** kidney, CD74, d-dopachrome tautomerase (d-DT/MIF-2), macrophage inhibitory factor, inflammation, Fabry, diabetes, polycystic kidney disease

## Abstract

CD74 (invariant MHC class II) regulates protein trafficking and is a receptor for macrophage migration inhibitory factor (MIF) and d-dopachrome tautomerase (d-DT/MIF-2). CD74 expression is increased in tubular cells and/or glomerular podocytes and parietal cells in human metabolic nephropathies, polycystic kidney disease, graft rejection and kidney cancer and in experimental diabetic nephropathy and glomerulonephritis. Stressors like abnormal metabolite (glucose, lyso-Gb3) levels and inflammatory cytokines increase kidney cell CD74. MIF activates CD74 to increase inflammatory cytokines in podocytes and tubular cells and proliferation in glomerular parietal epithelial cells and cyst cells. MIF overexpression promotes while MIF targeting protects from experimental glomerular injury and kidney cysts, and interference with MIF/CD74 signaling or CD74 deficiency protected from crescentic glomerulonephritis. However, CD74 may protect from interstitial kidney fibrosis. Furthermore, CD74 expression by stressed kidney cells raises questions about the kidney safety of cancer therapy strategies delivering lethal immunoconjugates to CD74-expressing cells. Thus, understanding CD74 biology in kidney cells is relevant for kidney therapeutics.

## CD74

CD74 (MHC class II invariant chain, Ii) is a transmembrane glycoprotein that regulates protein trafficking and is a cell surface receptor for the cytokines macrophage migration inhibitory factor (MIF) and d-dopachrome tautomerase (d-DT/MIF-2) (1). During kidney injury, leukocytes and intrinsic renal cells express CD74 (2). In the kidneys, MIF promotes experimental glomerular injury and cystogenesis, while recent reports suggest a protective role in interstitial fibrosis (2–4). However, CD74 is a multifunctional protein. Thus, the consequences of therapeutically targeting CD74 may differ from the consequences of targeting MIF. We, now, summarize the function of CD74 and review its expression and role in kidney injury, highlighting open questions. The detailed description of the role of CD74 in the immune system is beyond the scope of this review.

### CD74 functions

CD74 regulates intracellular trafficking and functions as a chaperone and a cell membrane receptor, modulating B, T, and dendritic cell responses (1, 5) and promoting tumor growth by increasing tumor cell survival or proliferation. Thus, CD74 is considered a therapeutic target in malignancy. In addition, CD74 regulates proliferation, survival, and secretion of inflammatory and fibrosis mediators in non-immune and non-tumor cells (1, 2). Thus, CD74 may modulate tissue injury and homeostasis beyond its effect on immune regulation.

#### Protein Trafficking

CD74 interacts with MHC class I and II proteins, contributing to antigen presentation. CD74 directs transport of MHC class II α and β chains from the endoplasmic reticulum (ER) or the cell surface to endosomes (6). As a chaperone, CD74 contributes to peptide editing in the MHC class II compartment. In endosomes, proteases degrade CD74, releasing MHC class II molecules. Prevention of CD74 degradation promotes the cell surface localization of MHC II. Extracellular CD74/cathepsin L complexes are found in human kidneys (1, 7, 8). CD74 also associates with angiotensin II type I receptors (AT1), leading to AT1 proteasomal degradation (9).

#### Cell Surface Receptor

Only a small amount of CD74 (2–5%) is expressed at the cell surface (10, 11). Cell surface CD74 is a receptor for MIF and MIF-2. CD74 lacks signaling motifs, but may generate fragments with transcription factor activity and binds to signaling proteins, such as CD44. Ligand binding to CD74 leads to CD74 phosphorylation, endocytosis, regulated intramembrane proteolysis (RIP), and release of CD74 intracellular domain (CD74-ICD) into the cytosol that translocates to the nucleus and activates NF-κB (10, 12–14). RIP might occur in cultured vascular smooth muscle cells since γ-secretase activity was required for MCP-1 expression induced by activating anti-CD74 antibodies. However, the pathophysiological implications remain unclear since MIF did not increase MCP-1 in these cells (15).

Migration inhibitory factor trimers activate CD74, and CD74 alone is required for MIF binding (16). In addition, MIF can also engage chemokine receptors CXCR2, CXCR4, and CXCR7 (10, 17). Cells that express two receptors, e.g., CD74 and CXCR2, may be more responsive to MIF (17). MIF-induced CD74 signaling complexes with CXCR2, CXCR4, or CXCR7 promote chemokine expression and chemotaxis (17–19). CD44 is the signaling component of the MIF-CD74 receptor complex and recruits the Src tyrosine kinase to activate ERK1/2 in macrophages and Syk, Akt, and NFκB in B cells (20, 21). Thus, the receptor complex activates kinase cascades and transcription factors. Interestingly MIF, itself increases CD44 expression (22).

Migration inhibitory factor-2 is 30% homologous to MIF, also activates CD74 (23, 24) and is responsible for residual CD74-dependent chemotactic activity in MIF^−/−^ mice (17). MIF-2 binding to CD74/CD44 activates kinases, downstream proinflammatory pathways, and β-catenin (25). However, MIF-2 lacks the pseudo(E)LR motif present in MIF that mediates interaction with CXCR2 and CXCR4, and thus, it is a more selective CD74 agonist than MIF. Circulating MIF-2 levels correlate with severity of sepsis, a cause of acute kidney injury, and MIF or MIF-2 blockade reduced systemic inflammation, protecting mice from lethal endotoxemia.

Migration inhibitory factor or MIF-2 activation of CD74 regulates cell survival and proliferation of B cells (21) and epithelial cells, including gastric epithelial cells and type II alveolar epithelial cells (26, 27). MIF or MIF-2 binding to CD74 protects the heart and liver from injury, including from ischemia–reperfusion (28–30). In the heart, CD74 promotes phosphorylation of the AMPK catalytic alpha subunit in response to increased intracellular calcium and activation of Ca^2+^/calmodulin-activated kinase kinase-2 (CaMKK-2) (29). Interestingly, liver expression of CD74 protected mice from lethality induced by agonistic anti-Fas antibodies as CD74 interfered with immediate early steps in Fas signaling at the plasma membrane, and the anti-CD74 antibody milatuzumab sensitized BJAB cells to Fas-mediated apoptosis (31). Fas ligand and Fas have long been known to promote kidney injury (32). In fact, agonistic anti-Fas antibodies induced glomerular cell apoptosis *in vivo* (33). Thus, CD74 interference with Fas signaling should be explored in kidney cells. The MIF/CD74/AMPK pathway also protects hepatocytes in metabolic liver injury, such as non-alcoholic steatohepatitis (30). In this regard, liver fibrosis was increased in MIF^−/−^ or CD74^−/−^ mice suggesting an antifibrotic effect of MIF/CD74 (34). Enhanced fibrosis was thought to result from the release of MIF inhibition of PDGF-induced migration and proliferation of hepatic stellate cells. MIF/CD74 also protects the lungs. Both MIF^−/−^ and CD74^−/−^ mice developed spontaneous emphysema by 6 months of age (35). However, CD74 may also contribute to disease, as discussed below for glomerulonephritis and kidney cysts. In this regard, CD74 deficiency reduced atherosclerosis in low-density lipoprotein receptor-deficient LDLR^−/−^ mice (36) and protected NOD mice from development of diabetes, probably by enhancing T regulatory cell number and impairing antigen presentation (37).

**Figure 1:**
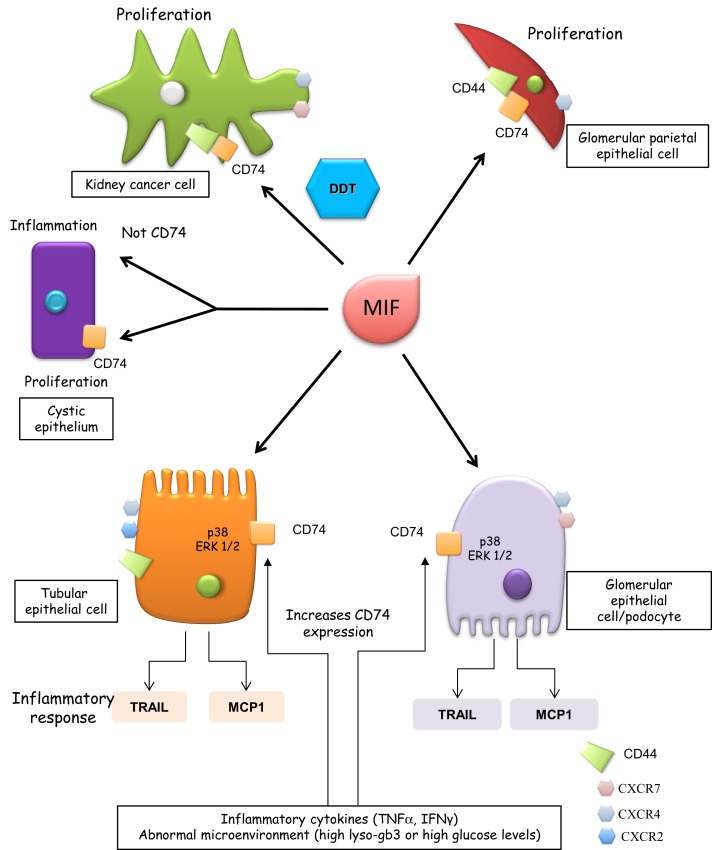
**CD74 functions in renal cells**. Glomerular parietal epithelial cells express CD44 when activated and it is thought that CD44 contributes to the proliferative response. CD44 is not expressed by podocytes and its role of CD74 signaling in tubular cells has not been characterized. Thus, in tubular cells, CD44 is not depicted as part of the CD74 signaling complex. Cells expressing CXCR2, CXCR4, and CXCR7 are also indicated, although these receptors are depicted away from MIF when in that specific cell type, there is no information on their involvement in MIF signaling. In tubular epithelium with genetic defects in PKD1, MIF promotes tubular cell proliferation and cystogenesis and a CD74 antibody blocked the MIF-induced phosphorylation of ERK but not inflammatory responses.

Among kidney cells, MIF induced proliferation in parietal epithelial cells but not in podocytes (4) (Figure 1). Absence of CD44 or the terminal differentiation state of podocytes may account for the differences. MIF, MIF-2, CD74, and CD44 promote clear cell renal cell carcinoma, cell proliferation, and HIF-activation (38, 39). While MIF and MIF-2 overlap in controlling cell survival and tumor formation, MIF-2 plays a dominant role in renal cancer tumor growth *in vivo* (40). MIF also confers resistance to senescence and cell death in mesenchymal stem cells through CD74-dependent AMPK-FOXO3a signaling and c-Met activation (41).

In renal tubular epithelial cells and podocytes, MIF binding to CD74 leads to persistent activation of p38 and ERK1/2 MAPK and expression of inflammatory mediators (e.g., TRAIL and MCP-1) (11, 42). MIF upregulation of inflammatory mediators was a late event, observed at 24 h (11). Thus, it was delayed as compared to responses elicited by the inflammatory cytokines TNF or TWEAK or metabolites, such as lyso-Gb3 (43, 44).

In summary, MIF-2 and MIF have an overlapping spectrum of activities mediated by CD74 activation and may cooperate, additively inducing chemokine secretion or survival in non-renal cells and proliferation in kidney cancer cells (45).

### Regulation of CD74 expression

CD74 expression is increased during tissue injury in diverse organs and in malignancies, including kidney cancer (2, 15, 28, 34, 46–48). There is limited information on the regulation of CD74 expression in renal cells. In normal mouse and human kidneys, tubular but not glomerular epithelium expresses low levels of CD74 (4, 11). By contrast, cultured human podocytes and proximal tubular cells and murine glomerular parietal epithelial cells express CD74 (4, 11). Abnormally high concentrations of certain metabolites (e.g., glucose and lyso-Gb3) and inflammatory cytokines, such as TNF, increase CD74 expression in podocytes and/or tubular cells (11, 49). IFN-γ increases CD74 expression *in vivo* in kidney tubular epithelium and in endothelial cells of larger kidney vessels (50). The factors known to upregulate CD74 expression in kidney cells may be relevant for diabetic nephropathy, Fabry disease, and inflammatory conditions.

### Regulation of CD74 interaction with MIF

Endogenous factors or drugs interfere with MIF binding to CD74 or downregulate CD74 expression and a better understanding of these interactions may provide therapeutic tools to manipulate the system. Some agents targeting MIF prevent MIF binding to CD74. These include antibodies, RPS19 (ribosomal protein S19), a component of the 40S small ribosomal subunit that binds MIF and behaves as an endogenous blocker of MIF binding to CD74 (51) and the small molecule MIF antagonist 3-(3-hydroxybenzyl)-5-methylbenzooxazol-2-one (MIF098) that decreases tautomerase activity and MIF-CD74 binding. MIF098 attenuated MIF-dependent ERK1/2 phosphorylation in human synovial fibroblasts (52) and promoted hyperoxia-induced lung injury *in vivo* (53), supporting the tissue-protective properties of MIF/CD74. Other compounds bind to CD74 or interfere with CD74 processing. The HLA-DRα1 domain binds to and downregulates CD74 on monocytes, directly inhibiting MIF binding to CD74 and blocking downstream inflammation in murine autoimmune encephalomyelitis. Adding a peptide extension [myelin oligodendrocyte glycoprotein (MOG)-35–55 peptide] that modified the secondary structure, enhanced the potency of the DRα1 domain in downregulating CD74 cell surface expression (54). Binding of partial MHC class II complexes comprised of linked β1α1 domains with covalently attached antigenic peptides [recombinant T-cell receptor ligands (RTLs)] to CD74 blocks the accessibility and availability of CD74 for MIF binding and downstream inflammation in monocytes (55).

The intramembrane protease presenilin homolog signal-peptide-peptidase-like 2a (SPPL2a) cleaves CD74. SPPL2a^−/−^ mice accumulate N-terminal fragments of CD74 that impair membrane traffic within the endocytic system and alter B cell biology (56, 57). Since in SPPL2a^−/−^ mice, CD74 signaling is inhibited, SPPL2a inhibitors may offer new pathways to inhibit CD74 signaling (58). Despite these recent advances, there is little or no information on the potential therapeutic or adverse effects of CD74 targeting strategies for kidney diseases and the available information is mainly derived from the study of CD74^−/−^ mice, which may have developed adaptive mechanisms over development.

The role of soluble CD74 is unclear. Circulating soluble CD74 with MIF neutralizing activity was increased in primary biliary cirrhosis and was hypothesized to contribute to liver fibrosis (59). Indeed, soluble CD74 ectodomain prevents binding of MIF to cell surface receptors (10). However, MIF/sCD74 complexes were found to enhance MIF antioxidant activity (60). Autoantibodies against CD74 (anti-HLA class II-associated invariant chain peptide, CLIP) were found in 67% of ankylosing spondylitis patients, in 15% of systemic lupus erythematosus patients, and only in 0.8% of blood donors (61, 62).

## CD74 and Kidney Injury

**Figure 2:**
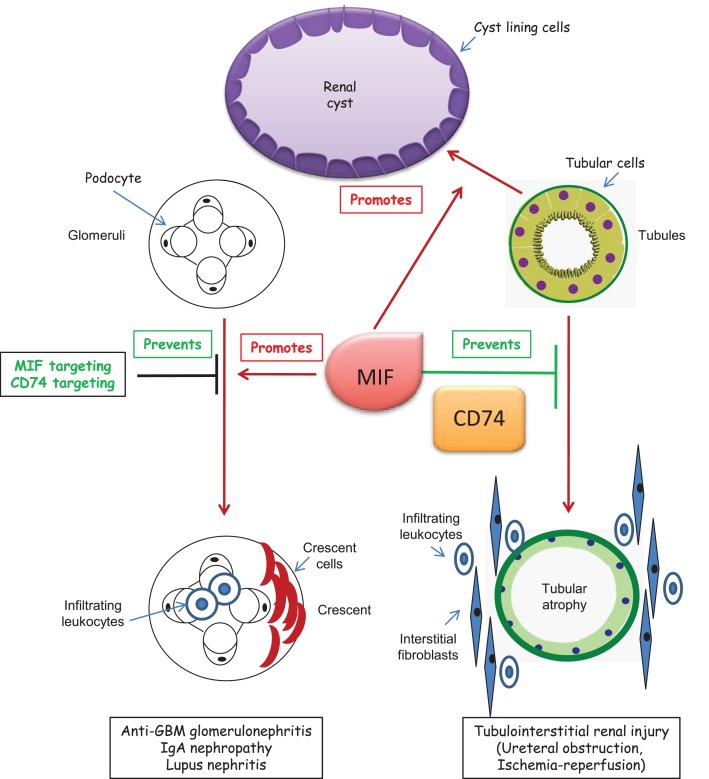
**CD74 in kidney disease**. Studies in CD74-deficient mice with kidney disease have only been published in abstract form. Thus, potential roles of CD74 in kidney disease have been mainly derived from abstracts or studies in which MIF was targeted in cultured cells or experimental animals. A putative effect of CD74 targeting on glomerular injury is only hypothetical and based on studies in which MIF was targeted. In polycystic kidney disease, MIF promotes cystogenesis. The role of CD74 is unclear, but CD74 expression is increased in cystic epithelium.

There is a growing body of evidence linking CD74 to promotion or protection from kidney injury (Figure 2).

### Expression of CD74 and related molecules in kidney injury

MIF has long been known to be upregulated in kidney injury (2). However, reports of increased CD74 expression are more recent and there is very little information on MIF-2.

Low CD74 expression may limit inflammatory and proliferative cell responses to MIF, and the increased renal cell CD74 expression observed during kidney injury (discussed below) may contribute, together with increased ligand availability, to elicit biological responses during kidney disease. Thus, overexpression of CD74 led to upregulation of NF-κB-dependent genes encoding cytokines in macrophages (63) and to NFκB activation and proliferation in human embryonic kidney cells (64).

CD74 is upregulated in tubular epithelial cells in at least some forms of chronic kidney disease (CKD), such as kidney graft rejection, diabetic and Fabry nephropathy and autosomal dominant polycystic kidney disease (ADPKD), and in podocytes in human diabetic nephropathy and Fabry nephropathy (11, 65, 66). A transcriptomics analysis also revealed increased kidney CD74 mRNA expression in human hypertensive nephropathy (11). CD74 is also increased in human clear cell renal cell carcinoma (67), in B cells, and kidney of lupus mice in parallel to progression of inflammation (68) and in the tubulointerstitium in anti-GBM nephritis, where it was expressed *de novo* in glomerular parietal epithelial cells and crescents (51). Interestingly, kidney cancer may be a complication of CKD-related acquired polycystic kidney disease (69). There is much less information on the expression of CD74 in acute kidney injury.

Migration inhibitory factor was recently identified as an important regulator of cyst growth and therapeutic target in ADPKD (3). MIF accumulated in cyst fluid of human ADPKD, promoted cystic epithelial cell proliferation and regulated apoptosis. In experimental murine polycystic kidney disease, MIF was required for renal inflammation and cyst expansion. CD74 expression was increased in *Pkd1* mutant renal epithelial cells and ADPKD kidneys, suggesting that MIF actions may imply CD74 activation. However, a CD74 antibody blocked the MIF-induced phosphorylation of ERK but did not modulate the MIF-induced inflammatory response of increased MCP-1 expression in culture (3).

The CD74 signaling machinery may also be upregulated in kidney disease. CD44 is rapidly upregulated after injury in acute and chronic experimental and human kidney disease including glomerulonephritis, diabetic nephropathy, cyclosporine nephrotoxicity, urate nephropathy, and ischemia–reperfusion kidney injury (70–76). Increased CD44 expression was localized to tubules, glomerular parietal epithelial cells, mesangial cells and infiltrating macrophages, T cells, and neutrophils (70, 72, 75–78).

Finally, the expression of at least some CD74 ligands is also increased in kidney injury. MIF expression is upregulated in acute and chronic, human, and experimental kidney diseases (2). However, there is no information on MIF-2 expression and kidney disease.

### CD74 targeting and kidney injury

Neutralizing anti-MIF antibodies, small molecules or endogenous inhibitors or MIF^−/−^ mice have shown that MIF aggravates anti-GBM glomerulonephritis (69–71), experimental IgAN (79), and lupus nephritis (80). More recently, the inhibitor of MIF/CD74 interactions RPS19 prevented the development of glomerular crescents, necrosis, inflammation, renal dysfunction, proteinuria, and the upregulation of MIF and CD74 in anti-GBM glomerulonephritis (51). However, MIF absence from mice did not protect from renal allograft rejection (81) or ureteral obstruction-induced kidney injury (82).

There is limited experimental and no clinical data on CD74 targeting in kidney injury. Given the multiple MIF and CD74 functions, studies are needed that explore whether targeting CD74 is therapeutic in models in which targeting MIF was beneficial. Information on the role of CD74 as a potential therapeutic target in tissue injury is mainly derived from CD74^−/−^ mice. As discussed above, CD74 deficiency may be beneficial for some non-renal diseases, such as liver and heart disease, and deleterious in others, such as vascular injury. Specifically, liver fibrosis was increased in CD74^−/−^ mice (34). An initial report found no protection from ureteral obstruction-induced kidney inflammation or fibrosis (82), but an abstract indicated that MIF targeting promoted interstitial fibrosis and inflammation following ureteral obstruction, whereas recombinant MIF reduced fibrosis (4). According to abstract reports, CD74 deficiency was also associated with increased interstitial fibrosis and inflammation following ureteral obstruction (day 5) and ischemia–reperfusion (day 21) (4). By contrast, CD74^−/−^ mice are protected from glomerular injury induced by anti-GBM antiserum (Djudjaj JASN2015).

Since MIF or MIF-2 activation of CD74 is tissue protective in heart ischemia and liver injury induced by activation of the Fas receptor or metabolic disorders, a similar protective effect maybe hypothesized in ischemic acute kidney injury or metabolic kidney diseases characterized by increased CD74 expression, such as diabetic nephropathy or Fabry disease. Interventional studies should test these hypotheses and evaluate whether changes in fibrosis after kidney ischemia–reperfusion is secondary to an improved initial kidney injury or to a specific action of fibrosis mechanisms. These studies should differentiate between complete abrogation of CD74 expression (CD74^−/−^) and therapeutic downregulation of CD74. Incomplete blockade of the system may have different consequences than complete CD74 absence, given the potential proinflammatory effects of excess CD74 activation.

### Kidney safety of lethal anti-CD74 immunoconjugates

The anti-CD74 antibody hLL1 milatuzumab, alone or as an immunoconjugate, is undergoing clinical trials to treat malignancy (83, 84). Milatuzumab binds to CD74 and promotes internalization of the antibody-CD74 complex, thus delivering conjugated antitumoral agents inside tumor cells with high CD74 expression, but not to normal cells with low CD74 levels (85). Since CD74 expression is also increased in renal cells from injured kidneys, nephrotoxicity is a potential complication of antitumoral anti-CD74 therapy, especially in patients with prior kidney disease since the active chemotherapeutic agent may be released inside already injured, CD74-expressing renal cells. This may be of special concern for one of the indications under study, multiple myeloma, which frequently causes kidney disease.

## Summary and Conclusions

The role of CD74 in kidney injury has barely been explored and the scarce information available is derived from CD74-deficient mice that may not recapitulate the findings of targeting CD74 *de novo* in an adult. CD74 may contribute to or protect from tissue injury in a disease-specific manner. Thus, like MIF, CD74 may protect from experimental kidney interstitial fibrosis but promotes glomerular injury, while MIF (and potentially CD74) also promotes polycystic kidney disease. A few years ago, the situation was less complex. Given the longstanding preclinical evidence for a pathogenic role of MIF in glomerular kidney disease, anti-MIF strategies were tested in renal disease, although the sponsor decided to terminate a phase 1 trial of the anti-MIF monoclonal antibody imalumab in lupus nephritis (NCT01541670). However, MIF, MIF-2, and CD74 may be tissue protective or promote injury in an organ- and disease-specific manner and different forms of therapeutic manipulation of the system may be envisioned for different indications, from inhibiting to actually activating CD74 signaling. The potential impact of future intervention on CD74 for kidney disease is double. On one hand, therapeutic modulation of the system may be used to treat kidney disease. On the other, the kidney may suffer adverse effects from the therapeutic targeting on non-kidney diseases. Therapeutic approaches blocking MIF or CD74 signaling for non-renal indications may theoretically promote kidney fibrosis as an adverse effect, while therapeutic approaches activating the system may cause or aggravate glomerular injury. Thus, a better understanding of CD74 and the kidney is required for nephrologists and non-nephrologists. Future areas of research include the potential therapeutic interest of molecules aimed at increasing or decreasing CD74 activity for different forms of kidney disease, including acute and chronic kidney injury, the potential renal adverse effects of these approaches when used for non-renal indications, the impact on kidney disease of naturally occurring circulating soluble CD74 or CLIP and the kidney safety of antitumor therapeutic strategies delivering toxins into CD74-expressing cells or targeting CD74. Additional unknowns to be solved include the drivers and consequences of CD74 RIP in kidney cells, the significance for kidney disease of MIF-2 and of CD74 actions on mesenchymal stem cells, and whether AMPK is activated by CD74 in renal cells since, contrary to the heart, no difference in AMPK activation by acute ischemia was observed between MIF^−/−^ and wild-type mice in the kidney, and this was attributed to lower CD74 expression in the kidney (86).

## Conflict of Interest Statement

The authors declare that the research was conducted in the absence of any commercial or financial relationships that could be construed as a potential conflict of interest.
